# Developing the Sus-Health Index: a combined measure for describing environmental impact and nutritive value of foods and meals

**DOI:** 10.1098/rstb.2024.0160

**Published:** 2025-09-18

**Authors:** Vasilis Grigoriadis, David Livingstone, Eva-Leanne Thomas, Paul Brereton, Jayne Woodside, Anne Nugent, Beatrice Smyth, George Hutchinson, Jelena Vlajic, Francisco Areal Borrego, Orla Collins, Novieta Sari, Rao Fu, Lynn Frewer

**Affiliations:** ^1^ Department of Economics, University of Ioannina, Ioannina, Greece; ^2^ Institute for Global Food Security, Queen's University Belfast, Belfast, Northern Ireland, UK; ^3^ School of Biological Sciences, Queen's University Belfast, Belfast, Northern Ireland, UK; ^4^ Centre for Public Health, Queen's University Belfast, Belfast, Northern Ireland, UK; ^5^ School of Mechanical and Aerospace Engineering, Queen's University Belfast, Belfast, Northern Ireland, UK; ^6^ Queen's Business School, Queen's University Belfast, Belfast, Northern Ireland, UK; ^7^ Northumbria University, Newcastle upon Tyne, Tyne and Wear, UK; ^8^ School of Natural and Environmental Sciences, Newcastle University, Newcastle upon Tyne, Tyne and Wear, UK

**Keywords:** food intake, nutrition, food labelling, food choice, sustainability assessment, food system

## Abstract

There is a need to develop a commonly agreed approach that describes a food’s nutritive value and environmental impact that can be used to inform consumer choice. A new single index, the ‘Sus-Health Index’, is presented. The index was co-created in conjunction with a range of stakeholders drawn from policy, industry and consumer representation groups. The index is a product of existing nutrition and environmental indices, using a composite indicator methodology, and can be presented quantitatively as a numeric value or qualitatively on a categorized, colour coded, scale ‘A (best)–E (worst)’. The index was applied to a dataset of food products and meals, and its sensitivity and applicability were assessed using a Monte-Carlo simulation. The results indicate that the Sus-Health scale represents a pragmatic indicator of a food or meal’s combined nutritional and environmental value and could be used to help inform consumer food choices.

This article is part of the theme issue ‘Transforming terrestrial food systems for human and planetary health’.

## 1. Introduction

Radical changes are needed in our food system to reduce its negative impact on the environment and improve dietary-related health. At present, the food system produces over 20% of global anthropogenic greenhouse gas emissions, while food production creates approximately 78% of global eutrophication and 32% of terrestrial acidification [1]. Failing to reduce, or indeed allowing acceleration of, these impacts will adversely affect natural ecosystems and the ecosystem services they deliver, drive climate change, biodiversity losses and reduce ecological resilience [2,3]. Furthermore, despite the global food system’s capability to feed more people [4,5], nutritional insufficiency is a serious issue with diets often lacking essential micronutrients such as vitamin A, iron, iodine and zinc [6–8]. In addition, excessive energy intake, alongside limited energy expenditure has led to rising levels of obesity and diet-related ill-health [9–11]. This is impacted directly by the food system both in the UK and globally [12,13] and is expected to continue in the absence of effective interventions [14].

It is important to transition towards more nutritious and sustainable diets [15–19]. However, it is unlikely that food business operators will change supply and production to more healthy and sustainable foods without evidence of a significant increase in consumer demand for such products. Consumers tend to place greater emphasis on nutrition than ‘sustainability’, but for some consumers, there is a lack of clarity regarding the term sustainability [20]. For example, the United Nations Food and Agriculture Organization provides a broad definition encompassing environmental impact together with a range of other socio-cultural attributes (e.g. economically fair and affordable) [21].

Implementing effective policy levers which simultaneously address nutrition and sustainability will catalyse change across the agrifood sector [22]. However, food policy with respect to the labelling of nutrition information is much more mature, and indeed standardized and mandatory, than labelling in relation to sustainability information [23]. For example, within the UK, nutrition policy with respect to food labelling is underpinned by the ‘Traffic-light Scheme’ [24], and the Ofcom Nutrient Profiling Model (UK NPM) [25]. The latter was developed for advertising food on television but now also underpins nutrition labelling of foods within ‘front of pack nutrition’ labelling guidelines. Other alternative NPMs and their relevance to policy have been suggested and reviewed by Labonté *et al.* [26]. By contrast, policy regarding sustainable food is relatively immature. Within the UK, the National Food Strategy Report [27] and the Committee on Climate Change recommend diet and lifestyle changes needed to reach net zero targets. However, sustainability considerations are not formally included in UK Government dietary guidelines and any labelling is voluntary. While sustainability labels on foods are being introduced, mainly focussing on environmental sustainability, for example Eco-score [28], it is unclear whether this will facilitate or further confuse consumer decision-making regarding healthy and sustainable food choices.

The aim of this research was to co-create and demonstrate, a pragmatic index, describing a meal/food’s nutritive and sustainability value and assess its performance on a range of meals, foods and ingredients. Recent reviews on the rationale and design of dual, joint and/or single indexes [29,30], have concluded that a robust and validated combined measure is not currently available. Further, such a measure needs to align with the preferences and requirements of consumers from different socio-cultural backgrounds, and who have access to different levels of economic resources. Although dual approaches have been presented [31], the development of a single index combining nutritive value and sustainability is relatively novel [30]. Given that many consumers complain of information overload in relation to labels [32], we hypothesized that a single combined index could be more effective than a dual approach. This reflects individual preferences or differences in prioritization, as consumers may value improved nutrition over enhanced production sustainability or vice versa [33]. This research is part of a larger study that includes additional work packages, and related findings have been submitted for publication [34–36] .

## 2. Methods

### (a) Co-creation of the combined index with stakeholders

The process of co-creation involves collaboration between multiple stakeholders for designing, implementing and measuring the outcomes of applied research, including within the area of health [37], healthier food retail environments [38] or sustainable agricultural practices [39]. During the co-creation process, different stakeholders and end-users collaborate and synthesize different kinds of knowledge, resources and competencies to solve a shared problem [38,40,41]. In the context of this work, the aim of the stakeholder group meetings were to:

(i) identify knowledge about the data needed to calculate the sustainability and nutrition components of the index from across the stakeholder constituency;
(ii) develop common approaches to understand how to weight and aggregate this knowledge in the development of the nutrition and sustainability components of the Sus-Health Index; and
(iii) consider and integrate the diversity of views across the stakeholder group (SG) regarding how the information might be displayed as a label on menus and products.

To do this three expert working groups (WGs) were formed:

— working group A (WGA) to identify an appropriate NPM;
— working group B (WGB) to identify an appropriate environmental impact model; and
— working group C (WGC) to decide on the weighting, combining and presentation of the index.

The WGs were overseen by a SG with representatives from private and public sector organizations.

### (b) Nutrition and environmental data sources and calculations

The quantities of nutrients/food components per portion were retrieved from McCance and Widdowson’s composition of foods integrated dataset [42] using Nutritics software [43] which was also used to convert the quantities in recipes (e.g. teaspoon, cup, etc.) to quantities in grams per portion, to find the quantities after cooking, and finally, to determine the losses of nutrients during cooking.^1^

**Figure 1.**
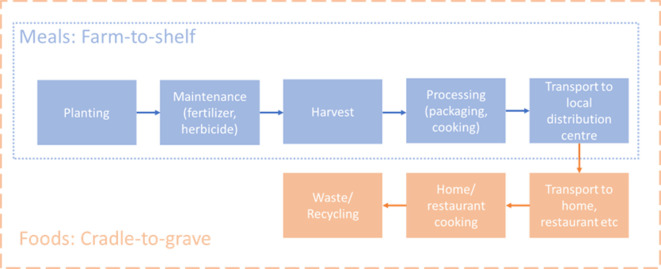
System boundaries for estimating the European Food Environmental Footprint Single Index of foods and meals.

For the environmental impacts, life cycle assessment (LCA) methodology was applied. LCA is considered as a standard and robust method for assessing the environmental impacts of products or services and is one of the most frequently used methods for evaluating food products [29]. For the LCA, the Agribalyse 3.1 [44], Agri-footprint 6.3 [45], WFLDB [46] and Ecoinvent 3.1 [47] databases were used. The Agribalyse and Agri-footprint databases primarily focus on French and Dutch food production, respectively. The WFLDB and Ecoinvent data bases provide a more globalized inventory of food production practices. Impact assessment calculations were carried out using Simapro 9 software [48] using the environmental footprint method produced by the European Commission [49]. The system boundary for the LCA was farm-to-shelf for meals and cradle-to-grave for individual food items (figure 1).

### (c) Sensitivity analysis

Composite indicators are built under certain assumptions about the way components are combined to create a composite score. These assumptions produce uncertainties which can be investigated through uncertainty and sensitivity analysis. Specifically, 10 000 Monte–Carlo simulations were used to estimate the confidence intervals on the ranks of food products, and a global sensitivity analysis was used to determine the individual contribution of each individual input’s uncertainty [50]. Three key uncertainties were examined, namely:

— *the aggregation procedure*: the arithmetic mean used for the Sus-Health Index is compared with the geometric mean. In general, the geometric mean tends to be lower than the arithmetic mean, especially when there are large variations in the data [50];
— *the normalization procedure:* the min-max method of the Sus-Health Index is compared with the *z*-score method (normalization with same mean and variance) [50]; and
— *the weighting procedure*: for the sensitivity analysis, the indicator’s weights are allowed to vary randomly within ±25% of their initial values.

## 3. Results

### (a) Co-creation activities

A SG was formed of interested parties from industry, policy, consumers, researchers and civil society. The SG was formed initially through direct contacts within the wider research team *pre-proposal,* and subsequently grew through additional promotion by the members themselves and in addition to large and small businesses included national (e.g. Food and Drink Federation), regional (e.g. Food NI) and local (e.g. council) representation. The SG met three times (every six months) and provided guidance and feedback to the research team and three expert WGs. Three meetings of each WG (nine in total) were held over an 18-month period during 2022−2023 (table 1).

**Table 1.** Summary of co-creation outputs with stakeholders. (S–H, Sus-health; WGA, working group A; NPM, nutrient profiling model; WGB, working group B; EFSI, European Food Environmental Footprint Single Index; GWP, global warming potential; WGC, working group C; NRF, Nutrient Rich Food; SAIN, nutrient density score; LIM, Limited nutrient score.)

name of working group	objectives	representatives of which constituent sectors	outcomes
stakeholder group	oversee development of S–H Index	industry, government (policy, food control, health), consumer associations, researchers	set 2 year timeframe, need to be pragmatic, cognisant of current policy developments, including both quantitative and qualitative presentation. Focus on environmental impact rather than wider sustainability that would be difficult to measure
WGA	identify appropriate nutritional component model	industry, government, researchers, consumers	identified Ofcom NPM as preferred model, presented on a semi-qualitative scale using Nutri-Score cut-offs. Others assessed: Nutrient Rich Food 9.3, SAIN (nutrient density score), LIM (Limited nutrient score)
WGB	identify appropriate environmental component model	industry, government, researchers, consumers	boundaries should be farm to shelf. Identified EFSI (Enviroscore) as the most appropriate model. Others assessed: GWP, Foundation Earth indicator
WGC	identify appropriate index weighting, calculation and presentation	industry, government, researchers, consumers/civil society	agreed on an equal (1 : 1) weighting of nutritive value to environmental impact using a composite indicator approach

A key issue raised by both industry and policy stakeholders was that the Sus-Health Index should, wherever possible, integrate good *existing* practice and, in terms of the nutritional component, be cogniscant of current policy. The situation in relation to the sustainability component was more complex as there are few agreed procedures, metrics or standards and policy is immature with few international agreements on standardized approaches. It was agreed that the focus should be on environmental impact rather than wider ‘sustainability’ for which many components, such as animal welfare and biodiversity, are not routinely measurable at present. Against this background the SG also decided that a ‘best endeavours’ and pragmatic approach should be used in producing the index within the timeframe of the activity (2 years). The Sus-Health Index was also to be developed in a way in which data sources could be updated and improved in the future.

#### (i) Nutritional component

Experts within WGA identified the UK NPM as the most appropriate nutritional component of the Sus-Health Index. Although alternative models for assessing nutritive value were considered (e.g. SAIN (nutrient density score), LIM (Limited nutrient score) [51], Nutrient Rich Food 9.3 (NRF9.3) [52]), these were not taken forward as they were considered unlikely to replace current nutrition labelling policy in the UK (or the European Union (EU)).

The NPM [25] is based on a points system considering seven nutrient and food components (energy, saturated fat, total sugar, sodium, percentage of vegetables, fruits or nuts, fibre and protein). The overall score has ranges from −15 to 40, where lower scores indicate healthier dietary patterns and a food is classified as ‘less healthy’ where it scores four points or more. A limitation of the UK NPM is the binary nature in which it categorizes foods as either less or more healthy. The working group therefore decided to use the cut-offs considered for the Nutri-Score model [53], which is based on UK NPM, uses the same scoring method, and is recommended in France and other EU countries.

Nutri-Score uses a five-level system [53] of A–E (table 2) and combines this classification with a traffic light system, ranging from dark-green A (highest nutritional quality) to dark-red E (lowest nutritional quality). This makes it a useful tool for communicating the healthiness of foods and meals to consumers. The combination of using the UK NPM values with the Nutri-Score cut-offs provided a means to provide both quantitative and qualitative indices for the nutritive components of the Sus-Health Index, something the expert WG considered to be highly valuable if it could be combined with a similar presentation of the environmental impact.

**Table 2.** Sus-Health cut-offs derived from the combination of European Food Environmental Footprint Single Index and the Nutrition Profiling Model Nutri-Score.

EFSI			Nutri-Score			Sus-Health	
cut-offs	normalization	letters	cut-offs	normalization	letters	normalization	letters
<4.00 × 10^−4^	0.02	A	−1	≤0.25	A	≤0.14 [14]^a^	A
≥4.00 × 10^−4^	0.02	B	0	≥0.27	B	≥0.15 [15]	B
≥1.45 × 10^−3^	0.08	C	3	≥0.33	C	≥0.20 [20]	C
≥2.00 × 10^−3^	0.11	D	11	≥0.47	D	≥0.29 [29]	D
≥1.00 × 10^−2^	0.56	E	19	≥0.62	E	≥0.59 [54]	E

^a^ Sus-Health scores can be presented as whole numbers between 0 and 100 to improve communication to consumers.

#### (ii) Environmental component

The European Food Environmental Footprint Single Index (EFSI) [55] was identified by WGB to be used for the environmental component of the Sus-Health Index. EFSI is a validated and verified methodology developed by Ramos *et al.* [55]. The EFSI combines 13 measurable impact categories^2^ normalized and weighted (electronic supplementary material, table S1) into a single index and was chosen as it presents a more holistic environmental impact compared to the global warming potential which is only one environmental impact category.

Furthermore, EFSI categorizes results into a five-scale score from A to E (table 2), called the ‘Enviroscore’ [55]. The Enviroscore combines the alpha score classification with a traffic light system, ranging from dark-green A (very low environmental impact) to dark-red E (very high). The combination of using the EFSI values with the Enviroscore cut-offs allowed the results of the Sus-Health Index to be displayed both quantitatively and qualitatively and enabled the results to be combined with the nutritive component of the index.

#### (iii) Combining the components using a composite indicator approach

To combine the environmental impact with the nutritional content of foods into one score the method of ‘composite indicator’ was used and involved three major steps: (i) normalization, (ii) aggregation, and (iii) sensitivity analysis [56]:

(i) *normalization* converted the scores into the same scale, for example 0−1, so they could then be aggregated using a simple normalization equation:
(3.1) $$X^{\prime }=\frac{X-X_{min}}{X_{max}-X_{min}};$$
(ii) *aggregation* combined the multiple indicators into one value. Many aggregation methods involve some kind of *weighting*, i.e. coefficients that define the relative weight of the indicators in the aggregation. Here, the arithmetic mean of the normalized EFSI and NPM (EFSI′ and NPM′) was applied (i.e. a 1 : 1 weighting):
(3.2) $$Sus\text{-}Health=0.5\times EFSI^{\prime }+0.5\times NPM^{\prime };\;and$$
(iii) *sensitivity analysis* quantifies the uncertainty in the scores and rankings of the composite indicator, and identifies which assumptions are driving this uncertainty. Three sources of uncertainty were examined; the aggregation method, the normalization method and the weighting.

Using the normalization and aggregation methods, the Sus-Health cut-offs were produced (table 2). Specifically, the combined cut-offs were calculated using the relevant bands from Nutri-Score (which is based on UK NPM) and the Enviroscore (which is based on EFSI). For Nutri-Score, the maximum value is 40 with a minimum of −15. For the Enviroscore, the minimum score is 0 but no maximum score is defined. Of the foods assessed by Ramos *et al.* [55] beef scored the highest with a mean EFSI value of 1.15 × 10^−2^ and highest value at 1.6 × 10^−2^. We therefore propose a theoretical maximum of 1.8 × 10^−2^ to cover any higher scores found in future research. An example of how the A band cut-off was calculated for the Sus-Health Index can be seen in equations (3.3)–(3.5):


(3.3) $$normalized\;Enviroscore\;A\;cut\text{-}off=\frac{3.9\times 10^{-4}-0}{1.8\times 10^{-2}-0}=0.02,$$

(3.4) $$normalized\;Nutri-Score\;A\;cut\text{-}off=\frac{-1-(-15)}{40-(-15)}=0.25,$$

(3.5) combinedSus−HealthAcut-off=0.5×0.02+0.5×0.25=0.14.

This procedure was repeated for the five Enviroscore and Nutri-Score cut-offs resulting in five Sus-Health scoring categories from A, for the best-performing foods (foods to encourage), to E, for the worst performing foods (foods to discourage).

### (b) Application and data sources

#### (i) Foods and meals

The Sus-Health Index was applied to two datasets: (i) individual food products and (ii) meals. The individual foods dataset enabled the index to be tested across a wide spectrum of scores (environmental and nutritional). A sensitivity analysis of the index was applied to this dataset. The meal dataset explores how the index can be used in a more realistic example of food choice environments, such as its application to a restaurant or catering environment.

**Figure 2.**
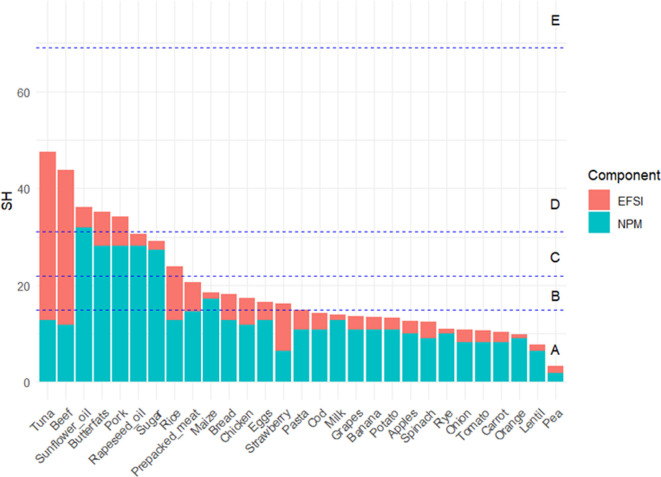
Relative components of the Sus-Health (SH) score for 29 foods.

The food dataset comprised the 29 example products and ingredients assessed in Ramos *et al.* [55] (figure 2) which are representative of the European market. For the environmental component, the EFSI and Enviroscore of these food products were extracted from Ramos *et al.* [55] (electronic supplementary material, table S2).

The meals dataset includes four very common home-cooked meals: chicken curry, vegetarian curry, spaghetti carbonara and beef stew, chosen as they represent a variety of protein and carbohydrate sources and are commonly consumed. Meal recipes were retrieved online from Tesco Real Food for the chicken curry, vegetarian curry and spaghetti carbonara; and BBC Recipes for the beef stew.^3^ Ingredients of the meals were expressed in grams before cooking per portion (electronic supplementary material, table S3).

#### (ii) Application of the Sus-Health Index to the dataset

The Sus-Health scores for individual food products (figure 2) ranged from 3.4 for peas (best score) to 47.6 for canned tuna (worst score). Fifteen food products were categorized in the A category; six in the B; three in the C; and five in the D category. No overall Es, or Es for the nutrition component were found in the dataset studied. Es for environmental impact were found for canned tuna and beef but the overall score was mitigated with a good nutrition score (electronic supplementary material, table S2). As a result, no food products were categorized in the E group for the combined Sus-Health score. Considering the contribution of each individual indicator to the Sus-Health score, the nutritional component is more prominent for most of the food products. Against this, the environmental component has a bigger influence on the beef, tuna and strawberry scores. Finally, the two components have a balanced influence on the combined index score for rice and peas.

#### (iii) Sensitivity analysis

**Figure 3.**
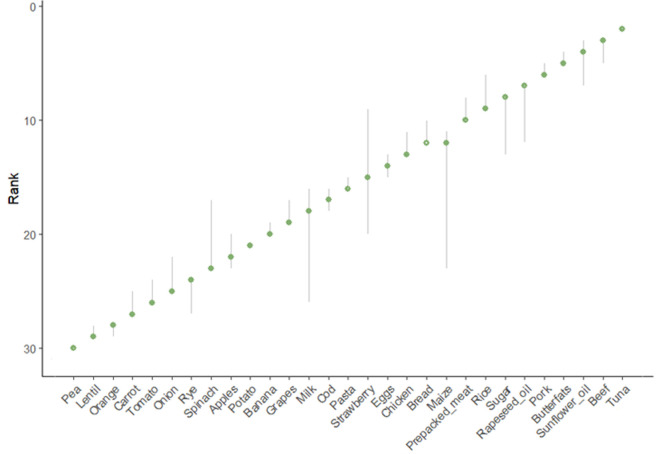
Figure shows 90% confidence intervals on ranks of the Sus-Health Index. Note: grey bars are the 90% confidence intervals, green point is the median rank across the uncertainty analysis. Food products are ordered according to their nominal rank (figure 2).

Sensitivity analysis was applied only to the food dataset (figure 3) as it represents a wider variety of scores than the four meals assessed. A narrow uncertainty interval indicates a more robust ranking, dependent only to a limited extent on the selection of assumptions. A wider interval indicates higher volatility of the food’s ranking, which markedly depends on the specific design of the Sus-Health Index [57]. For the Sus-Health Index, confidence intervals tend to be generally narrow, with an average variation of 2.83 ranking places across a total of 29 ranks. The most variable food products in the sample are maize, milk, strawberries and spinach. Their relevant quantile differences are 12, 10, 10 and 6, respectively.

For the global sensitivity analysis, using Monte–Carlo analysis and 10 000 repetitions [58], two variance-based indices are calculated: the *first-order index (*electronic supplementary material, figure S1), which estimates the individual contribution of each input to the output uncertainty, and the *total order index (*electronic supplementary material, figure S2), which estimates the contribution of each input including interactions with other inputs [58,59]. The results found that the most sensitive component of the Sus-Health Index is the aggregation method followed by the normalization method, followed by the weighting. Therefore, the Sus-Health Index is robust in relation to the equal weighting method selected, but quite sensitive to the methods of aggregation and normalization used.

#### (iv) Sus-Health Index applied to four meals

**Figure 4.**
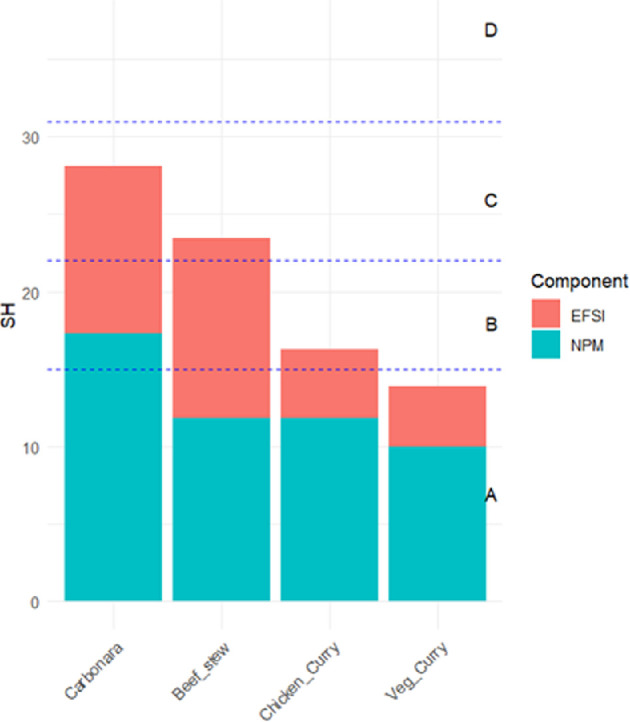
Relative components of Sus-Health (SH) score for four meals.

Considering the Sus-Health scores for the four meals (figure 4); the vegetable curry has the best (lowest) score of 14, followed by chicken curry with 16, beef stew at 24 and carbonara with the worst score of 28. Considering the contribution of each component to the Sus-Health Index, for chicken curry and vegetarian curry, the nutritional component has almost double the input to the score than the environmental component, indicating a low environmental impact for both meals. For carbonara, the components have similar contributions to the scores, while for beef stew, the environmental component has a higher contribution to the Sus-Health Index, owing to the high environmental impact of beef production. Categorizing the Sus-Health scores within the five-category range, vegetable curry falls into the A category, chicken curry into the B category, and beef stew and carbonara into the C category.

#### (v) Qualitative presentation of Sus-Health Index

**Figure 5.**
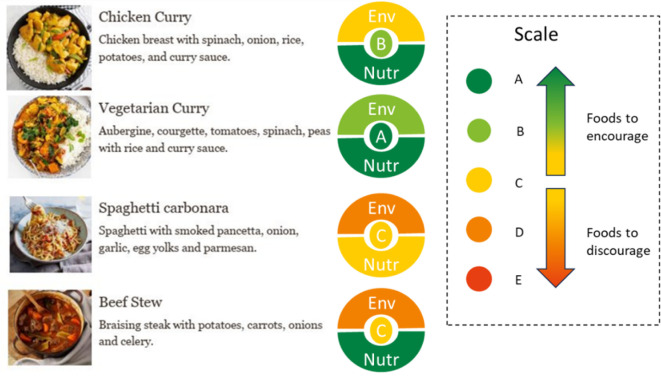
Visualization of the Sus-Health scores using the chosen ‘doughnut’ presentation.

In addition to the quantitative scale produced for the Sus-Health Index, which would be useful for industry in formulation/reformulation activities, stakeholders also identified the need for a qualitative label to describe the index values in a user-friendly manner to consumers. Several versions with various combinations of quantitative and qualitative representations were presented (electronic supplementary material, figures S3–S5) to the SG, with that shown in figure 5 being the preferred label. The label displays the A–E scales and traffic-light colours as a total Sus-Health score in the centre with the individual environmental and nutritive value components in the surround.

## 4. Discussion

The combination of environmental impact and nutritive value of foods into a composite score provides simplicity and clarity for food system stakeholders, such as consumers, policymakers and industry professionals, and allows for quick comparison between foods or meals and easy communication of results. While some other recently published indices, such as the Sustainability Index [54], the Med Index Checklist [60] and the Comprehensive Assessment Index [61], include wider aspects of sustainability like economic and socio-cultural dimensions, for the purposes of food labelling, it is complex and can be potentially confusing to consumers [30]. In addition, the Med Index [60], which promotes adherence to the mediterranean diet, is a region-specific approach, limiting its global applicability. The Sus-Health Index uses LCA to assess environmental impacts of foods, a common approach implemented globally [29] (though the accuracy and relevance of LCAs are dependent on data availability and assumptions made in the analysis), and uses the NPM to assess nutritional value of foods, a model that has been adapted and applied internationally.

A systematic review into sustainable food profiling models, described as the scientific basis for the labelling of food products was undertaken by Bunge *et al.* [30] in 2021. They found that the aggregation into one score can introduce bias towards either the nutritional or environmental components [30]. Aggregation could also lead to a loss of detail and overlooking of nuances such as foods that have a large negative environmental impact but are nutritionally adequate, or vice versa, as well as weighting issues. While previous studies have taken a dual approach [31], excessive or complex labelling might result in ‘information overload’, diminishing the intended benefits. Buratto & Lotti [32] argue that choice overload reduces judgement accuracy, particularly for sustainability claims, emphasizing the importance of simple label designs. Similarly, Sunstein [62] warns that too much information can induce cognitive strain, distort judgements and lead to disengagement. By including colour-coded information, about the environmental impacts and nutrition, around the central aggregated score, the Sus-Health Index overcomes oversimplification and loss of detail and also displays the equal weighting of the composite score without overloading the consumer with information.

Despite the number and variety of approaches to creating combined food labelling assessing environmental impact and nutritive value [30,54,60,61], consumer preference for label design has not been considered and future work on this is required. Work regarding this area is currently underway and due for publication [35]. Future work could also look at the impact of the visualization as developed in the study, in case for example, a food scores a dark green A on nutritional quality and dark red E on environmental impact.

An important factor determining the impact of combined score labelling on the food system is the extent to which consumers will use the label to make food choices. Indeed, the usefulness of food product label information depends on consumers' trust in that information [63]. Consumer trust in labels is determined by perceptions that the label information is underpinned by expert analysis and that the information conveyed by the label is based on accurate and transparent underpinning scientific information [64], third-party verification [65,66] or traceability of specific product attributes, such as the use of pro-ecological production methods [67]. The information conveyed by the label must also be understandable and salient and/or important to the consumer [68,69]. This work attempts to provide a robust and simplified index that provides useful easy to understand information to consumers.

There is a need to test the impact of such an index on consumer choices, particularly for those consumers from lower demographic groups who make poor choices with respect to healthy sustainable diets and have been found to ignore current food labels such as the traffic light system currently used [69]. Research is already underway to test the impact of including Sus-Health indices (with label) on consumer real-world meal choices within a range of quick service restaurants using a living laboratory approach. Similarly, studies are planned to assess a range of other presentation modes and index label styles on consumer behaviour on a large scale and across different regulatory contexts.

Nutritional and particularly environmental policies are dynamic and evolving. Any index that attempts to combine the two elements, and be exposed to consumers, will have limitations related to the current state of the art with regards to research *and* policy. The SG were cognizant of this evolving landscape and were keen to put clear boundaries on the research for the development of the index in terms of time, policy and scope (of sustainability) and applicability. A key aim was that while the index should have a qualitative presentation with colour coding for ease of use for consumers, it should also be able to be presented in a (semi) quantitative manner. This enables industry to benchmark their products through this new ‘lens’, allowing them to formulate/reformulate their products in the future as appropriate. It is for this reason that the index developed within this work has both a quantitative and qualitive element and presentation. As nutritional and environmental policies evolve, the index must prioritize clarity and usability to enhance and promote consumer engagement.

The immature nature of sustainability policy and supporting standardization (not least of terms) resulted in a narrower definition of ‘sustainability’ being used for the equation. Wider sustainability components that encompass additional societal factors such as modern slavery, animal welfare, etc., that are not easily measurable and do not easily lend themselves to a quantitative index, were not included. As a result, the ‘sustainability’ component of the index can be viewed as solely comprising ‘environmental impact’.

Another key issue when composing the index, outside of the choice of the nutrition and environmental models, is what weighting to apply to each. For the Sus-Health Index, a 1 : 1 ratio was chosen, but alternative ratios could be argued. The feeling within the stakeholder group was that the choice of ratio could vary depending on a range of factors such as age, societal background, wealth and so on. As a result, a pragmatic choice of 1 : 1 was made. There is a gap in the literature, which requires further study, regarding how consumers may differentially weigh nutritional and sustainability information in their decision-making, whether a combined measure is acceptable (and indeed understood), and whether this further interacts with the socio-cultural factors influencing food choices.

A limitation of the current work is the relatively small number of foods assessed in the Monte–Carlo simulation. While there is no specific rule about the minimum number of samples required, the 29 foods used in this study is similar to the rule of thumb of approximately 30 observations. As a result, the variability of the measures used in sensitivity analysis (e.g. rank order) could be higher compared to a larger sample (owing to increased susceptibility to outliers or extreme values). Expanding the selection of foods within the same category (e.g. including beans in addition to lentils within legumes) as well as incorporating foods from additional categories (e.g. desserts) could increase the representativeness and reduce the sensitivity of the Sus-Health Index.

Furthermore, while the work reported here focuses on the rationale and composition of the index, there is a need to expand the dataset to include the complete spectrum of A–E values. While E values for the environmental impact were found (canned tuna and beef) no overall Es were found at the time of reporting, nor Es for the nutrition component. This may be a result of our focus on main meals as opposed to starters and desserts, some of which, owing to high fat, sugar and/or salt may well provide an E score for the nutritive component of the Sus-Health index. Expanding the dataset assessed by the Sus-Health Index is a key step for future research.

It is also important not to neglect economic trade-offs of improving Sus-Health scores such as the impacts of dietary transitions on employment in the agrifood sector, and on the affordability of food [70]. The relevance of this information in relation to the combined index will be assessed within future research within the Sus-Health project.

## Data Availability

Data files are provided as the electronic supplementary material [71].

## Footnotes

^1^
Nutritics run online on https://www.nutritics.com/en/.

^2^
Climate change; ozone depletion; ionizing radiation; photochemical ozone; respiratory inorganics; acidification (terrestrial and freshwater); eutrophication (fresh water; marine; terrestrial); land use; water scarcity; resource use (energy carriers; minerals and metals).

^3^
Find the online links on https://realfood.tesco.com/recipes.html and https://www.bbc.co.uk/food/recipes, respectively.
